# Triose-phosphate isomerase deficiency is associated with a dysregulation of synaptic vesicle recycling in *Drosophila melanogaster*

**DOI:** 10.3389/fnsyn.2023.1124061

**Published:** 2023-02-28

**Authors:** Aelfwin Stone, Oliver Cujic, Angel Rowlett, Sophia Aderhold, Emma Savage, Bruce Graham, Joern R. Steinert

**Affiliations:** ^1^Division of Physiology, Pharmacology and Neuroscience, School of Life Sciences, University of Nottingham, Nottingham, United Kingdom; ^2^Division of Computing Science and Mathematics, Faculty of Natural Sciences, University of Stirling, Stirling, United Kingdom

**Keywords:** triose-phosphate isomerase, glycation, neurodegeneration, synaptic release, vesicle pool, neuromuscular junction, *Drosophila*

## Abstract

**Introduction:**

Numerous neurodegenerative diseases are associated with neuronal dysfunction caused by increased redox stress, often linked to aberrant production of redox-active molecules such as nitric oxide (NO) or oxygen free radicals. One such protein affected by redox-mediated changes is the glycolytic enzyme triose-phosphate isomerase (TPI), which has been shown to undergo 3-nitrotyrosination (a NO-mediated post-translational modification) rendering it inactive. The resulting neuronal changes caused by this modification are not well understood. However, associated glycation-induced cytotoxicity has been reported, thus potentially causing neuronal and synaptic dysfunction via compromising synaptic vesicle recycling.

**Methods:**

This work uses *Drosophila melanogaster* to identify the impacts of altered TPI activity on neuronal physiology, linking aberrant TPI function and redox stress to neuronal defects. We used *Drosophila* mutants expressing a missense allele of the TPI protein, M81T, identified in a previous screen and resulting in an inactive mutant of the TPI protein (*TPI^M81T^*, wstd^1^). We assessed synaptic physiology at the glutamatergic *Drosophila* neuromuscular junction (NMJ), synapse morphology and behavioural phenotypes, as well as impacts on longevity.

**Results:**

Electrophysiological recordings of evoked and spontaneous excitatory junctional currents, alongside high frequency train stimulations and recovery protocols, were applied to investigate synaptic depletion and subsequent recovery. Single synaptic currents were unaltered in the presence of the wstd^1^ mutation, but frequencies of spontaneous events were reduced. Wstd^1^ larvae also showed enhanced vesicle depletion rates at higher frequency stimulation, and subsequent recovery times for evoked synaptic responses were prolonged. A computational model showed that TPI mutant larvae exhibited a significant decline in activity-dependent vesicle recycling, which manifests itself as increased recovery times for the readily-releasable vesicle pool. Confocal images of NMJs showed no morphological or developmental differences between wild-type and wstd^1^ but TPI mutants exhibited learning impairments as assessed by olfactory associative learning assays.

**Discussion:**

Our data suggests that the wstd^1^ phenotype is partially due to altered vesicle dynamics, involving a reduced vesicle pool replenishment, and altered endo/exocytosis processes. This may result in learning and memory impairments and neuronal dysfunction potentially also presenting a contributing factor to other reported neuronal phenotypes.

## Introduction

During the pathology of neurodegenerative diseases, redox stress mediated by oxidative and nitrergic species is one of the hallmarks of disease, and is responsible for pathological pathways augmenting neurodegeneration (Calabrese et al., 2010; Gu et al., 2010; Sbodio et al., 2019). A major contributory factor enhancing cellular redox stress is prolonged microglia activation and neuroinflammation, associated with nitric oxide (NO)-mediated post-translational changes causing aberrant protein modifications (Gu et al., 2010; Steinert et al., 2010; Bradley and Steinert, 2016; Hannibal, 2016). In Alzheimer’s disease, the NO-mediated post-translational modification induced by peroxynitrite, 3-nitrotyrosination (3-NT), of β-amyloid (Aβ) oligomers presents a direct pathway for increasing oligomer stability and toxicity (Kummer et al., 2011). We and others have identified triose-phosphate isomerase (TPI) as a target for NO-mediated post-translational modifications in neurodegenerative diseases, rendering the enzyme inactive and linking to enhanced glycation signalling (Guix et al., 2009; Tajes et al., 2014a,b; Bourgognon et al., 2021).

Triose-phosphate isomerase is a metabolic enzyme in the glycolytic pathway that catalyses the conversion and adjusts the equilibrium between glyceraldehyde 3-phosphate (GAP), and dihydroxyacetone phosphate (DHAP). GAP and DHAP are products of the cleavage of fructose 1,6-bisphosphate catalysed by aldolase. While DHAP has a role in fatty acid metabolism, GAP continues in the glycolytic pathway ultimately producing pyruvate. Although the isomerisation of DHAP to GAP is not required to produce pyruvate, it increases the efficiency of glycolysis (Roland et al., 2016).

Enhanced nitrergic stress facilitates 3-nitrotryrosination of TPI at tyrosine (Tyr)165 and Tyr209 which reduces its enzymatic activity (Guix et al., 2009) and leads to a shift of balance of the metabolites toward DHAP formation. Subsequently, DHAP transforms spontaneously into the cytotoxic metabolic by-product methylglyoxal (Tajes et al., 2014a,b). A nitro-oxidative environment thus favours high levels of 3-nitrotyrosinated TPI in Alzheimer’s disease (AD) (Guix et al., 2009), and as a result, inhibition of this enzyme leads to nonenzymatic methylglyoxal-mediated enhanced protein glycation (Ahmed et al., 2003; Guix et al., 2009), and formation of advanced glycation end-products (AGE). AGE formation has a causative role in the vasculature complications of diabetes mellitus and several neurodegenerative diseases, including Alzheimer’s, Parkinson’s, and Huntington’s diseases (Li J. et al., 2012; Yamagishi, 2019).

Triose-phosphate isomerase deficiency also causes a disease characterised by an autosomal, recessive mutation producing neurological symptoms alongside (most commonly neonatal) haemolytic anaemia and progressive muscular impairment which, in humans, often results in infant death due to respiratory failure (Orosz et al., 2009). Although anaemia is characteristic of glycolytic enzymopathies, the severe neurologic impairments are unique to TPI deficiency.

Physiologically, TPI homodimerises for full catalytic activity, however the conformational changes due to mutations cause dissociation of the dimers (Seigle et al., 2008; Roland et al., 2015). This results in a less active monomer and misfolded protein, even though pyruvate production or energy metabolism at the system level are not affected (Orosz et al., 2009).

There are direct links between glycation signalling and synapse dysfunction. A biochemical study revealed that glycation of α-synuclein reduces its binding to synaptic-like vesicles thus impacting on fusion (Uceda et al., 2022). α-Synuclein promotes the fusion of vesicles with the plasma membrane, it is involved in the homeostasis of synaptic vesicles during the neurotransmission (Vargas et al., 2014), and it is able to regulate release of vesicle from the presynaptic pools (Zadali et al., 2019; Yoo et al., 2022), rendering this pathway as a candidate for causing synaptic dysfunction in mammalian neurons. There is general consensus that AGEs are responsible for synaptopathologies, AGEs causes a suppression of hippocampal long-term potentiation (Zhang et al., 2014), memory impairments (Li X. H. et al., 2012) and indirect evidence suggests that the receptor for AGE (RAGE) contributes to synaptic dysfunction seen in AD (Criscuolo et al., 2017). However, the exact underlying mechanisms and extend of AGE mediated pathology remain largely unknown.

In *Drosophila*, an initial characterisation of TPI dysfunction revealed a reduced longevity without showing altered bioenergetic or mitochondrial phenotypes (Celotto et al., 2006; Gnerer et al., 2006). This model investigated the effects of TPI deficiency in flies lacking the functional protein (wstd^1^ (Gnerer et al., 2006)). Although synaptic phenotypes in other TPI mutants (*TPI^M80T^*) in *Drosophila* have not been reported, more recent studies suggested that a *Drosophila* TPI mutation (*dTPI^T73R^*) induces changes in synaptic vesicle dynamics as illustrated by altered FM1-43 loading kinetics (Roland et al., 2016).

We utilised *Drosophila* to assess the effects of a functionally deficient TPI (*TPI^M81T^*) protein at the level of synaptic activity, behavioural phenotypes and longevity. This study identified a reduced vesicle replenishment at higher rates of activity without impacting on low frequency calcium-mediated evoked release. This data is supported by a computational model showing a reduction of available vesicles during high frequency trains, resulting in an apparent increased rate of depletion. In contrast, spontaneous calcium-independent release is disturbed by TPI deficiency resulting in lower frequencies of miniature events. Furthermore, we identified learning and memory impairments in those larvae and confirmed a reduced life span, all providing new information on the neuronal effects caused by TPI dysfunction.

## Materials and methods

### Fly husbandry

Flies were raised on standard maize media at 25°C at a 12 h light-dark cycle. Backcrossed wstd^1^ flies were kindly provided by Prof. Barry Ganetzky, University of Wisconsin (Gnerer et al., 2006). The homozygote w*^1118^* line was used as a control. All data presented are comprised of experiments in male and female *Drosophila* as our data revealed no sex differences (data not shown).

### Odour-associative learning

The odour-associated learning paradigm was used as reported previously (Weiglein et al., 2019). Innate preferences were tested first by placing larvae in a petri dish with the experimental odour on one side, leaving larvae to crawl for 3 min and recording larval position on the dish. For paired training, cohorts of 10–15 larvae were placed at the centre of a Petri dish (9 cm inner diameter) filled with 1% agarose, supplemented with fructose (2 M) as a taste reward (+) which included an odour-containing filter paper (*n*-amylacetate (AM), diluted 1:20 in paraffin oil). Paraffin has no behavioural significance as an odour (Saumweber et al., 2011). For training, larvae were placed at the midline and free to move for 3 min at a dish containing AM filter paper at each of the opposing edges of the Petri dish and fructose-containing agar (AM+). Subsequently, they were transferred to a Petri dish without fructose which had two non-AM filter papers (EM), and they were left there for the same amount of time. After such AM+/EM training sessions, they were transferred to the centre of a test Petri dish, where an AM odour filter paper was presented on one side and were thus tested for their preference for AM. After 3 min, the numbers of larvae (#) on the AM side, on the EM side, and in a 10-mm wide middle zone were counted. Larvae crawling up the sidewalls of the Petri dish were counted for the respective side, whereas larvae on the lid were excluded from the analysis. A preference (Pref) was calculated:


$$P\,r\,e\,f=\frac{\#\,\mathrm{AM}-\#\,\mathrm{EM}}{\#\,\mathrm{total}}$$

Preference indices range from +1 to −1, with positive values indicating preference and negative values indicating avoidance of AM. Across repetitions of the experiments, in half of the cases the sequence was as indicated (AM+/EM), whereas in the other cases it was reversed (EM/AM+). The procedure for unpaired training was the same, except that the Petri dishes featured either only AM or only the reward. After such AM/EM+ training (again in half of the cases the sequence was reversed: EM+/AM), the preference test was carried out as above. From the Pref scores after paired and unpaired training, a Preference Index (PI) was calculated:


$$P\,I=\frac{\mathrm{Prefpaired}-\mathrm{Prefunpaired}}{2}$$

Performance indices range from +1 to −1. Positive PIs indicate appetitive associative memory, whereas negative values indicate aversive associative memory.

### Locomotor activity

Age-matched third instar larvae (∼120 h of age) were selected, washed and placed onto a moist, food-free surface at a constant temperature of 20°C. Crawling activities were imaged over 10 min using AnyMaze software v7.16 (Stoelting Co., Wood Dale, IL, USA) and data was analysed off-line as reported previously (Robinson et al., 2014).

### Electrophysiology

Two-electrode voltage clamp (TEVC) recordings were performed as described previously (Robinson et al., 2018; Spiers et al., 2019). Sharp-electrode recordings were made from ventral longitudinal muscle 6 (m6) in abdominal segments 2 and 3 of third instar larvae using pClamp 10.5, an Axoclamp 900A amplifier, and Digidata 1550B (Molecular Devices, USA) in hemolymph-like solution 3 (HL-3; Stewart et al., 1994). Recording electrodes (20–50 MΩ) were filled with 3 M KCl. All synaptic responses were recorded from muscles with input resistances ≥4 MΩ, holding currents <4 nA at −60 mV and resting potentials more negative than −60 mV at 25°C. Holding potentials were −60 mV. The extracellular HL-3 contained (in mM): 70 NaCl, 5 KCl, 20 MgCl_2_, 10 NaHCO_3_, 115 sucrose, 5 trehalose, 5 HEPES, and 1.5 CaCl_2_. Average single evoked excitatory junction potential (eEJC) amplitudes (stimulus: 0.1 ms, 1–5 V) were based on the mean peak eEJC amplitude in response to 10 presynaptic stimuli (recorded at 0.2 Hz). Nerve stimulation was performed with an isolated stimulator (DS2A, Digitimer). All data were digitised at 10 kHz and for miniature event recordings, 60-s recordings were analysed to obtain mean miniature EJC (mEJC) amplitudes. Both, mEJC and eEJC recordings were off-line low-pass filtered at 500 Hz and 1 kHz, respectively. Materials were purchased from Sigma-Aldrich (UK).

### Cumulative postsynaptic current analysis

The apparent size of the vesicle pools was probed by the method of cumulative eEJC amplitudes (Schneggenburger et al., 1999). Muscles were clamped to −60 mV and eEJC amplitudes during a stimulus train were calculated as the difference between peak and baseline before stimulus onset of a given eEJC. Receptor desensitisation was not blocked as it did not affect eEJC amplitudes, because a comparison of the decay of the first and the last eEJC within a train did not reveal any significant difference in decay kinetics (not shown). The size of release-ready pool was obtained by back extrapolating a line fit to the linear phase of the cumulative eEJC amplitude plot to last 300 ms of a 1000 ms train to time zero (50 and 60 Hz).

### Immunohistochemistry

Third instar larvae were dissected in ice-cold PBS then fixed in 4% paraformaldehyde. After permeabilisation with PBS-0.1% Triton (PBS-T) and blocking with PBS-T containing 0.2% bovine serum albumin (BSA) and 2% normal goat serum, larval fillets were incubated at 4 °C overnight in solutions of primary antibody. The following antibody dilutions were used: NC82 (supernatant) anti-Brp (Bruchpilot) 1:200. After 3 × 10 min washes in PBS-T, larvae were incubated with AlexaFluor 488 goat anti-HRP (Jackson Immuno Research) and AlexaFluor 546 goat anti-mouse 1:500 dilution for 90 min at room temperature. Larvae were mounted using Vectashield mounting medium (Vector Labs) and NMJ m6/7 (segments A2 and A3) images were acquired with a Zeiss laser-scanning confocal microscope (Zeiss LSM880C). Image analysis was performed using ImageJ software.

### Longevity

Groups of 10 newly emerged adult male and female flies were separated and transferred to new vials containing food and deaths were scored daily. Flies were transferred to new food twice a week and otherwise left undisturbed at a 12–12 h light-dark cycle. No significant gender differences were observed, and all presented data was pooled.

### Modelling

A simple model with activity-dependent vesicle recycling (Richardson et al., 2022) was used to fit eEJC amplitudes during trains of stimuli. In the model, vesicles in a releasable pool of normalised size n(t) may release with a fixed probability p(t) = p_*vr*_ on the arrival of a presynaptic action potential at time s to give an eEJC amplitude proportional to n(s)p(s) (Eq. 4). Vesicles in this releasable pool are replenished at a rate τr from a large (assumed effectively infinite) reserve pool (Eq. 1). In the absence of presynaptic action potentials, replenishment proceeds at a constant background rate (time constant τb). Following a presynaptic action potential, the replenishment rate is instantaneously raised to a higher rate (time constant τh; Eq. 3) which then decays back to the background rate with time constant τd (Eq. 2). The model equations are:


(1) $$\frac{d\,n}{d\,t}=\frac{1-n\,\left(t\right)}{\tau_{r}\,\left(t\right)}-\sum_{s}p\,\left(t\right).n\,\left(t\right).\delta \,\left(t-s\right)$$

(2) $$\frac{d\,\tau_{r}}{d\,t}=\frac{\tau_{b}-\tau_{r}\,\left(t\right)}{\tau_{d}}$$

(3) $$\tau_{r}\,\left(s\right)=\tau_{h}$$

(4) e⁢E⁢J⁢C⁢(s)=n⁢(s).p⁢(s)

The model is implemented in Matlab (The MathWorks Inc.). Differential equations are solved by simple forward Euler integration. Least squares fits to the data were carried out using the Matlab fminsearch routine.

### Statistical analysis

Statistical analysis was performed with Prism 9.4 (Graphpad Software Inc., San Diego, CA, USA). Statistical tests were carried out using an unpaired Student’s *t*-test, unless stated otherwise. One-way ANOVA test was used when applicable with a posteriori test (with Tukey’s multiple comparisons). Cumulative frequency distributions were compared using the Kolmogorov–Smirnov test (K–S test). After confirming normal data distributions (Shapiro–Wilk test) and homogeneity of variance (Bartlett’s test), we used parametric statistics. To compare single genotypes against a chance level, we used one sample *t*-test or Wilcoxon signed-rank test (odour learning). Cumulative survival curves are presented and compared using the Log-rank (Mantel-Cox) test. Data are expressed as mean ± SEM and box and whisker plots where *n* is the number NMJs, larvae and flies as indicated. Significance is shown as *p < 0.05, ^**^p < 0.01, ^***^p < 0.001 and ^****^p < 0.0001.

## Results

Wstd^1^ flies have previously been described as having neurological impairments showing enhanced temperature-sensitive paralysis and delayed recovery from paralysis without having compromised bioenergetics such as ATP levels (Gnerer et al., 2006; Roland et al., 2013, 2016). However, detailed observations associated with neuronal dysfunction or synaptopathology are not well established, although changes in vesicle dynamics have been implicated in larvae expressing mutant TPI (Roland et al., 2016).

### TPI deficiency induces functional alterations at the NMJ

**FIGURE 1:**
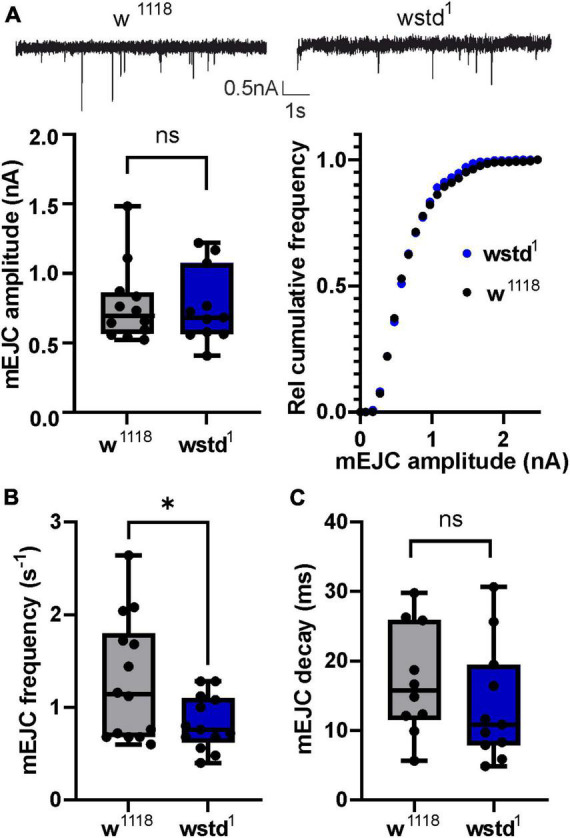
wstd^1^ does not affect single release events but reduces frequency of spontaneous release. (**A**, top) Representative mEJC recordings (top) from a w*^1118^* and wstd^1^ larva. (Below) Mean mEJC amplitudes (w*^1118^ n* = 13 and wstd^1^ *n* = 11, unpaired Student’s *t*-test, *p* > 0.05) and cumulative amplitude distributions (K–S test, *p* > 0.05). **(B)** Mean mEJC frequencies (events per second) for wstd^1^ and w*^1118^* (*n* = 10–13, *p < 0.05). **(C)** mEJC decay kinetics (*p* > 0.05, unpaired Student’s *t*-test).

To understand the physiological effects of TPI deficiency, we recorded spontaneous miniature excitatory junctional currents (mEJC) and evoked (eEJC) at the NMJ from wstd^1^ and w*^1118^* control 3*^rd^* instar larvae. Mean mEJC amplitudes were unaffected in wstd^1^ larvae [w*^1118^*: 0.8 ± 0.1 nA vs. wstd^1^: 0.8 ± 0.1 nA, *p* = 0.923 (*n* = 11–12) Figure 1A] which was confirmed by a more sensitive test plotting the relative cumulative amplitude distributions (*p* = 0.7066, K–S test). However, the frequency of mEJC events was reduced in wstd^1^ larvae [w*^1118^*: 1.3 ± 0.2 s^–1^ vs. wstd^1^: 0.8 ± 0.1 s^–1^, *p* = 0.0316 (*n* = 11–12) Figure 1B], with mEJC decay kinetics [tau: w*^1118^*: 17 ± 2 ms vs. wstd^1^: 14 ± 3 ms, *p* = 0.3443 (*n* = 13–14) Figure 1C] being unaffected. The data shows that single spontaneous release events are not affected by a TPI deficiency, however, the rate of spontaneous release is reduced, possibly indicating changes in calcium-independent vesicle fusion.

**FIGURE 2:**
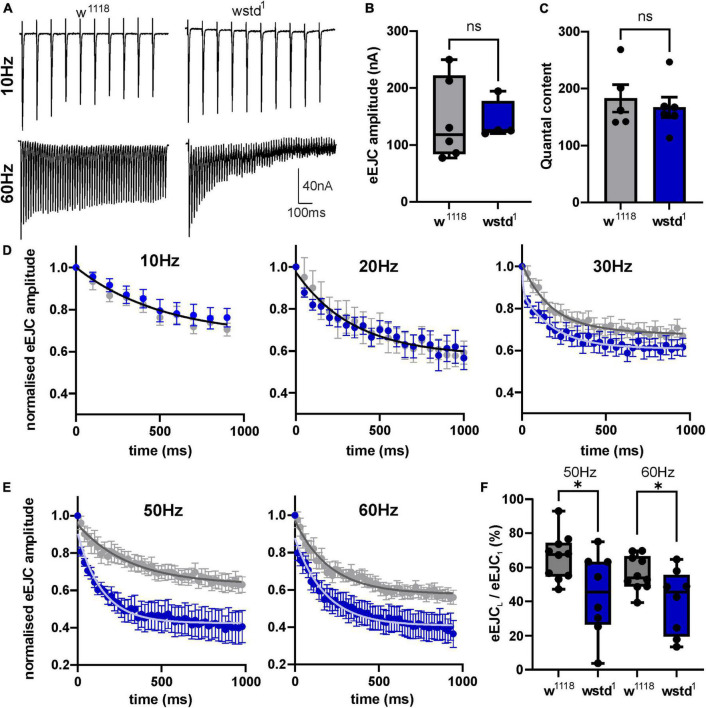
wstd^1^ larvae exhibit an enhanced depression during higher frequency trains of activity. **(A)** Representative recordings of synaptic trains at 10 and 60 Hz, note the increased rate of depression at 60 Hz in a wstd^1^ larva. **(B)** eEJC amplitudes of initial synaptic currents (*n* = 4–6, *p* > 0.05, unpaired Student’s *t*-test). **(C)** Quantal content for both genotypes as calculated by dividing the initial eEJC amplitude by the corresponding mEJC amplitude of the same NMJ (*p* > 0.05, unpaired Student’s *t*-test). **(D,E)** Averages of the relative eEJC amplitudes for each train frequency (mean ±SEM). Solid black line represents the single exponential fit to the decay of the amplitudes (10 and 20 Hz). At 30–50 Hz the decay of the amplitudes could be best fitted using functions with two different tau values. **(F)** Ratio of last to first eEJC amplitude within a 50 and 60 Hz train (*n* = 8–10, *p < 0.05, unpaired Student’s *t*-test).

To gain further insights into underlying mechanisms, we quantified evoked release. A comparison of synaptic currents to single stimuli and trains of activity ranging from 10 to 60 Hz for 1,000 ms was performed in both genotypes. Single initial eEJC amplitudes did not differ between the two groups [w*^1118^*: 143 ± 29 nA vs. wstd^1^: 141 ± 17 nA, *p* = 0.9551, (*n* = 5–6) Figures 2A, B], nor did the quantal content [w*^1118^*: 183 ± 24 vs. wstd^1^: 167 ± 18, *p* = 0.6055 (*n* = 5–6) Figure 2C] suggesting no change in calcium-mediated evoked release. Interestingly, despite having unchanged single evoked responses, the transmitter release during trains of higher activity was markedly altered in wstd^1^ larvae. Although at 10 and 20 Hz stimulus frequency, we did not notice any changes in rates of release as measured by a single exponential fit to the decaying amplitudes [w*^1118^* vs. wstd^1^: 10 Hz: *F*_(3, 184)_ = 1.096 *p* = 0.3523, 20 Hz: *F*_(3, 272)_ = 0.4438 *p* = 0.7218 (*n* = 8–10) Figures 2A, D], at 30 Hz and above, we noticed that the amplitudes decayed at a faster rate indicative of an augmented depression. Single exponential fits to normalised amplitudes differed between both genotypes [w*^1118^* vs. wstd^1^: 30 Hz: *F*_(3, 444)_ = 19.20 *p* < 0.0001, 40 Hz: *F*_(3, 354)_ = 256.8 *p* < 0.0001, 50 Hz: *F*_(3, 644)_ = 118.1 *p* < 0.0001, 60 Hz: *F*_(6, 1079)_ = 61.48 *p* < 0.0001 (*n* = 8–10) Figures 2A, D, E]. Table 1 summarises mean tau values following single exponential fits to amplitude decays at different stimulation frequencies. This enhanced depression can further be illustrated by calculating the ratio of the last to the first eEJC amplitude within the train for 50 and 60 Hz stimulus frequencies [eEJC_*L*_/eEJC_1st_: 50 Hz: *w*^1118^: 66 ± 4% vs. wstd^1^: 44 ± 8%, *p* = 0.0254 (*n* = 8–10), 60 Hz: w*^1118^*: 57 ± 3% vs. wstd^1^: 39 ± 6%, *p* = 0.0291 (*n* = 8–10) Figure 2F]. This data suggests that the wstd^1^ mutation only causes impairments on the overall calcium-stimulated evoked release of transmitter during longer periods of high activity. We tested an initial possibility which, amongst others, could explain such phenotype: vesicle pools are reduced in TPI deficient larvae which allows fewer available vesicles to fuse with the membrane and be released. This could potentially be a consequence of a reduced release and replenishment of vesicle pools in that, at faster rates of release, a slower endocytosis and replenishment limits the refill of pools resulting in a faster net depletion (Hallermann et al., 2010). Alternatively, the synapses could possess a higher release probability which would lead to a faster rate of depletion (Delgado et al., 2000) although an initially higher eEJC amplitude would be expected under those conditions which we did not observe.

**TABLE 1:** Tau values for exponential fits to the amplitude decay at different stimulation frequencies (mean ± SEM).

Frequency (Hz)	wstd^1^	*w* ^1118^
10	413 ± 42	377 ± 91
20	256 ± 99	327 ± 63
30	128 ± 36	206 ± 25
40	236 ± 76	321 ± 96
50	147 ± 30**	387 ± 75
60	148 ± 28**	256 ± 18

**p < 0.01.

**FIGURE 3:**
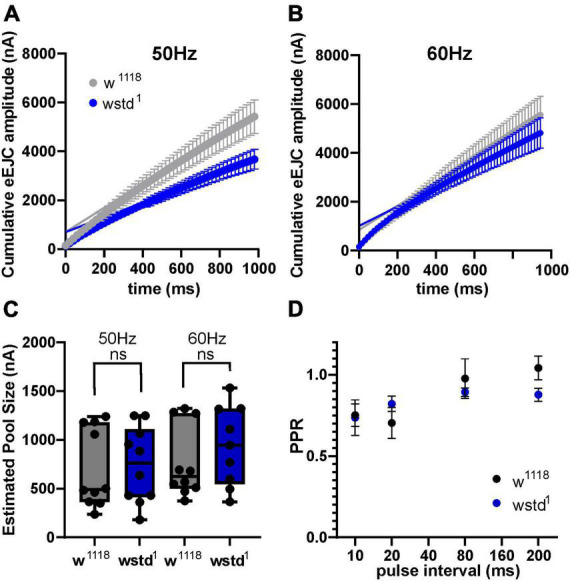
Estimated vesicle pool size is not affected by wstd^1^. **(A,B)** Mean cumulative eEJC amplitudes for 50 and 60 Hz trains for both genotypes. Solid lines indicate linear fits to the last 300 ms of the train, back extrapolated to time 0 s with the y-axis intercept providing an estimation for the vesicle pool size. **(C)** Estimated pool sizes for both genotypes for both frequencies (*p* > 0.05, unpaired Student’s *t*-test). **(D)** Paired pulse ratios (PPR) for both genotypes (*n* = 10 each, *p* > 0.05, unpaired Student’s *t*-test).

To test both possibilities we initially calculated available pool sizes. We determined the pool size using a method successfully applied at the *Drosophila* NMJ by analysing the cumulative total current of trains of higher frequency stimulation (Weyhersmuller et al., 2011). Stimulation at 50 Hz or above for >500 ms in 1.5 mM extracellular calcium retrieves vesicles from the readily releasable pool (RRP) (Kuromi and Kidokoro, 2005; Hallermann et al., 2010). The pool size (in nA) of wstd^1^ larvae was not different when assessed following a 50 and 60 Hz train of synaptic activities [50 Hz: w*^1118^*: 706 ± 127 nA vs. wstd^1^: 743 ± 122 nA, *p* = 0.8338, 60 Hz: w*^1118^*: 772 ± 117 nA vs. wstd^1^: 938 ± 136 nA, *p* = 0.3637 (*n* = 8–10) Figures 3A–C].

A change in paired pulse ratios (PPR) would indicate changes in initial vesicular release probabilities (p_*vr*_) at glutamatergic synapses (Holderith et al., 2012). We wanted to confirm whether p_*vr*_ was affected by TPI deficiency in wstd^1^ larvae by calculating PPR at 10, 20, 80, and 200 ms stimulus intervals. At all intervals, there was no difference in PPR between both genotypes [10 ms: w*^1118^*: 0.79 ± 0.07 vs. wstd^1^: 0.74 ± 0.11, *p* = 0.893; 20 ms: w*^1118^*: 0.71 ± 0.11 vs. wstd^1^: 0.82 ± 0.05, *p* = 0.474; 80 ms: w*^1118^*: 0.98 ± 0.15 vs. wstd^1^: 0.89 ± 0.03, *p* = 0.887; 200 ms: w*^1118^*: 1.1 ± 0.08 vs. wstd^1^: 0.88 ± 0.04, *p* = 0.899 (*n* = 6 each) Figure 3D] indicating that synapses from wstd^1^ larvae did not exhibit altered release probabilities although a tendency towards lower PPR values was noted in wstd^1^.

**FIGURE 4:**
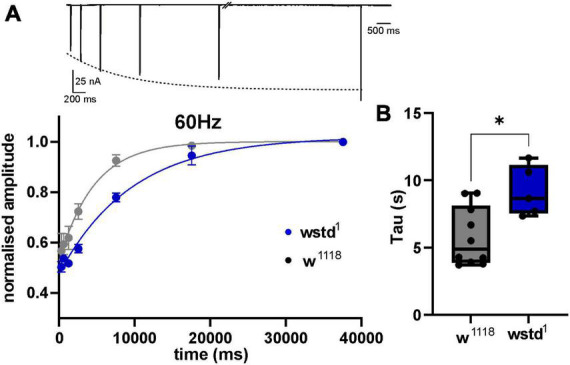
TPI deficiency slows down recovery of vesicle pools following high frequency trains. (**A**, top) Raw trace illustrating the recovery protocol: following a 60 Hz train (not shown), stimuli were applied at 640, 1,280, 2,560, 7,560, 17,560, and 37,560 ms past the last stimulus of the train. Dotted line represents an exponential fit to the eEJC amplitudes. (Bottom) Mean eEJC amplitudes normalised to the final recovery data point, fitted by a single exponential. **(B)** Decay tau values for both genotypes (*n* = 6–10, *p < 0.05, unpaired Student’s *t*-test).

To interrogate the possibility whether a diminished refilling of vesicle pools underlies the observed increase in depression, we performed recovery-from-depletion studies, and calculated time constants of recovery following application of higher stimuli frequencies which have been reported to deplete ready releasable vesicle pools (Hallermann et al., 2010). After a 1000 ms train of 60 Hz stimulation, w*^1118^* larvae showed a recovery of eEJC amplitudes with a time constant of 5.8 ± 0.6 s compared to wstd^1^ of 9.2 ± 0.8 s [*p* = 0.0107 (*n* = 6–10) Figure 4] and single exponential fits to the mean recovery data differed between both genotypes [*F*_(3,120)_ = 9.661, *p* < 0.0001 (*n* = 6–10)]. This data indicates that endocytosis may be compromised in wstd^1^ larvae.

Together, our data suggests that TPI deficiency diminishes vesicular release during periods of higher synaptic activity, such as at 30 Hz and above. As a result, the data suggest that release probability and pool size were not affected by TPI deficiency but point towards a slower replenishment of pools in wstd^1^ larvae.

### Modelling of release parameters confirms a reduced readily releasable vesicle pool under high frequency activity

**FIGURE 5:**
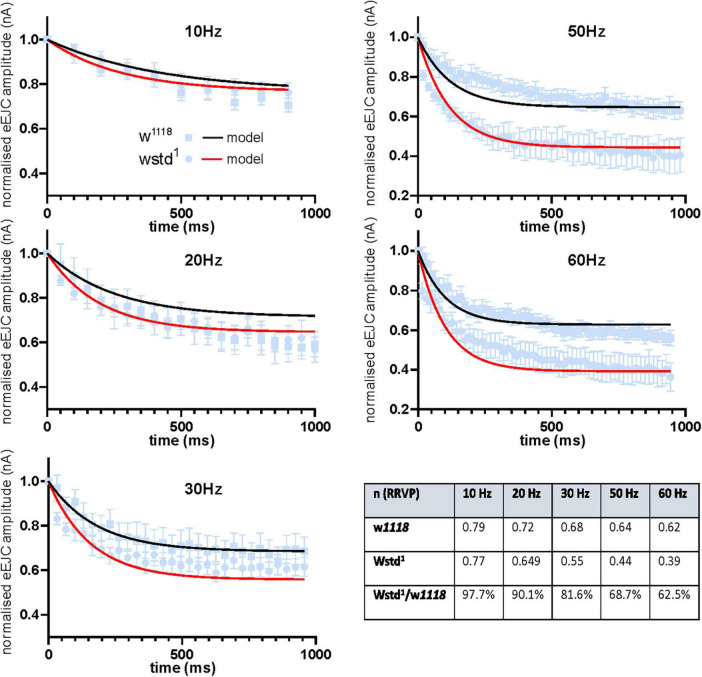
Modelled data show TPI deficiency induces and accelerated decline in vesicle availability during trains of enhanced activity. Experimental data of eEJC amplitudes are plotted for different frequencies (mean ± SEM) and are superimposed with model predicted curves for w^1118^ (black) and wstd^1^ (red) NMJs. The summary table shows the size of the normalised ready-releasable vesicle pool size (RRVP).

Fits for vesicle release probability (p_*vr*_) and activity-dependent vesicle recycling time constants (τh and τd) were obtained simultaneously across all 5 stimulation frequencies. Background vesicle recycling is not strongly relevant during fast stimulation and determines recovery following the end of the stimulus train, so background recycling time constants (τb) were set to the recovery time constants measured experimentally. Model fits to the data are shown in Figure 5. The fitted parameter values are summarised in Table 2.

**TABLE 2:** Computer modelled decay tau values.

	p_vr_	τ h (ms)	τ d (ms)	τ b (ms)
w*^1118^*	0.0593	23.9	165.3	5800
wstd^1^	0.0934	248.2	3078.2	9200

These values indicate a modest change in release probability, but a significant decline in activity-dependent vesicle recycling in the TPI larvae: the slower time constant of activity-dependent recycling in the TPI animals (TPI: τh = 248.2 ms vs. w*^1118^*: τh = 23.9 ms) results in less vesicle recovery between high-frequency stimuli (30 Hz and above), even with an apparently slower return to background recycling rates (decay time constants, TPI: τd = 3078.2 ms vs. w*^1118^*: τd = 165.3 ms). The fraction of the normalised ready-releasable vesicle pool (RRVP) available for the next stimulus at the end of each train at each stimulus frequency is shown in the following Table 3.

**TABLE 3:** Computer modelled of normalised ready-releasable vesicle pool (RRVP).

n (RRVP)	10 Hz	20 Hz	30 Hz	50 Hz	60 Hz
w*^1118^*	0.793	0.72	0.6862	0.6466	0.6284
wstd^1^	0.7749	0.649	0.5597	0.4439	0.3927
wstd^1^/w*^1118^*	97.7%	90.1%	81.6%	68.7%	62.5%

### TPI deficiency does not cause developmental or morphological changes at the NMJ

**FIGURE 6:**
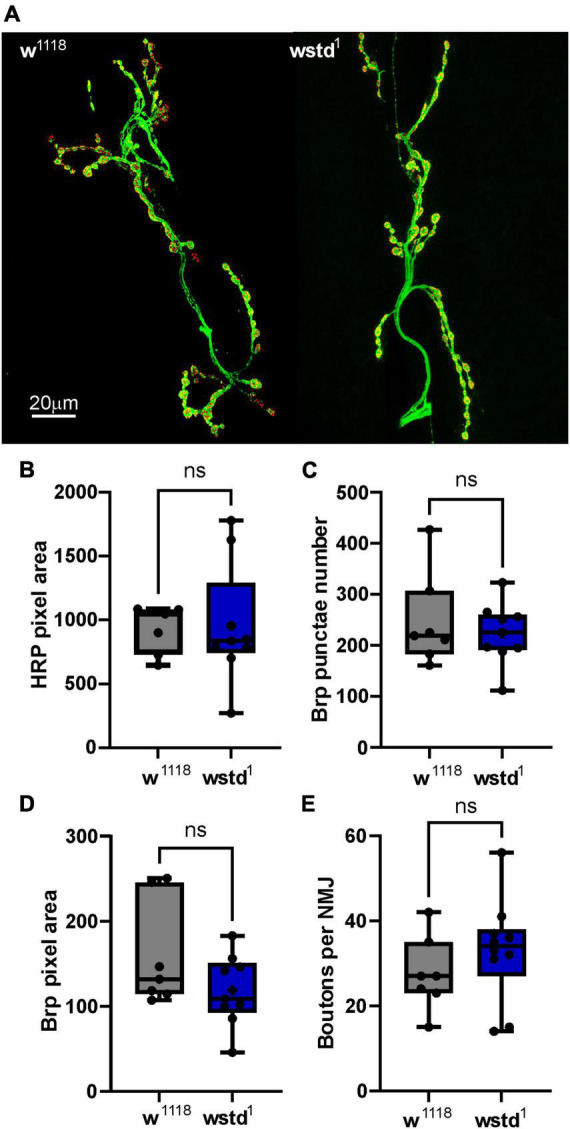
Immunocytochemistry of NMJs reveals no morphological changes in wstd^1^ larvae. **(A)** Representative confocal images of 3*^rd^* instar *Drosophila* larval NMJs from a w*^1118^* and wstd^1^ genotype (maximal projections, HRP-green, Brp-red). **(B)** Comparison of total HRP (NMJ) signal, number of Brp puncta **(C)**, total Brp signal **(D)**, and bouton numbers **(E)** (*n* = 7–8, *p* > 0.05, unpaired Student’s *t*-test).

As the electrophysiology studies point towards functional effects caused by TPI deficiency, we wanted to test whether any morphological changes at the NMJ may contribute to the observed phenotypes. The most prominent variable which potentially affects release of vesicle is the number and size of active zones which is determined by the expression and localisation of Brp (bruchpilot) (Banovic et al., 2010; Chou et al., 2020). We performed immunocytochemistry experiments to label the active zone protein Brp and neuropile using HRP to visualise the NMJ morphology (Figure 6). Maximal projections of z-stacked images were used to compare signals for Brp and HRP. Firstly, we counted Brp punctae as indication for the number of active zones. This measure did not reveal any differences between the two genotypes [w*^1118^*: 248 ± 35 vs. TPI: 224 ± 20, *p* = 0.537 (*n* = 7–9 NMJs) Figure 6B] and is in agreement with published data (Nijhof et al., 2016; Kopke et al., 2020). Next, we wondered whether the size of a single Brp signal would differ between the groups. This readout, however, did not reveal any differences [total area: w*^1118^*: 159 ± 23 a.u. vs. TPI: 118 ± 14 a.u., *p* = 0.921 (*n* = 7–9 NMJs) Figure 6C]. Finally, the total area of the NMJs or the numbers of boutons did not differ between genotypes [total area: w*^1118^*: 936 ± 70 a.u. vs. TPI: 954 ± 156 a.u., *p* = 0.142; number of boutons: w*^1118^*: 27 ± 3 vs. TPI: 33 ± 4, *p* = 0.326 (*n* = 7–10 NMJs) Figures 6D, E] suggesting that the mutant TPI caused no morphological changes at the synapse.

### Locomotor activity

**FIGURE 7:**
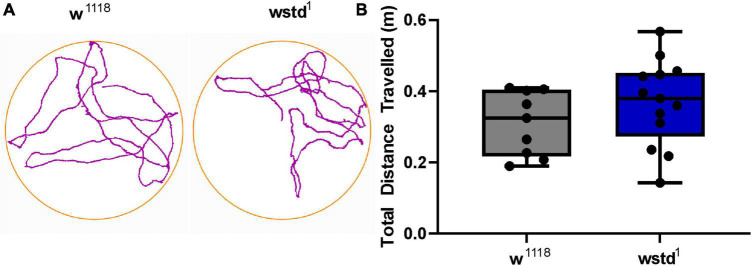
Locomotor activity is not affected by TPI deficiency. **(A)** Larval crawling activities were recorded over 10 min within a crawling arena and tracks were recorded and plotted using AnyMaze software. **(B)** Average activities show no differences between both genotypes (*n* = 9–13, *p* > 0.05, unpaired Student’s *t*-test).

In order to investigate if TPI impairment impacts on whole larval locomotor activity, we performed crawling assays where larvae were placed onto a moist surface and movements were tracked for 10 min to assess low frequency neuromuscular transmission and muscle contractions. To test this behaviour, larvae were placed on a crawling device allowing on-line monitoring of individual larval activities (Steinert et al., 2006). Locomotive impairment is a commonly used diagnostic for characterisation of models for neurodegeneration where foraging behaviours are affected (Jakubowski et al., 2012). Both genotypes performed equally well during the 10 min recording period with no locomotor impairments detected in TPI deficient larvae relative to w*^1118^* control larvae [distance travelled: w*^1118^*: 0.31 ± 0.03 m vs. wstd^1^: 0.37 ± 0.03 m, *p* = 2322 (*n* = 9–13) Figure 7] suggesting that the low frequency (∼1 Hz) synaptic excitation is sufficient to support locomotor behaviour.

### Learning and memory

**FIGURE 8:**
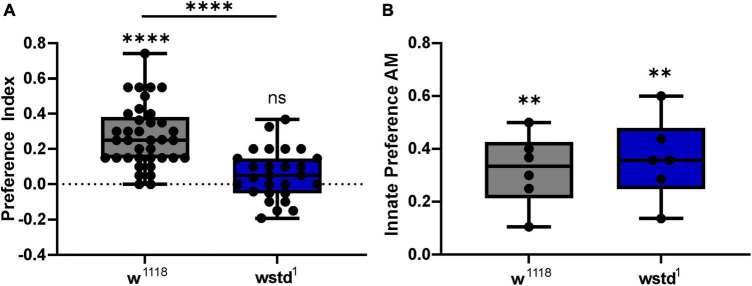
TPI deficiency causes odour associative learning deficits. **(A)** Preference index (PI) was calculated for 3*^rd^* instar *Drosophila* larvae of both genotypes. PIs were significantly reduced in wstd^1^ larvae (unpaired Student’s *t*-test, *p* < 0.05). **(B)** Innate preferences to AM did not differ between genotypes (unpaired Student’s *t*-test, *p* > 0.05, however, both lines exhibited a similar innate attraction to AM (*n* = 28–40, *p* < 0.05, one sample *t*-test).

*Drosophila* larvae can associate odours with gustatory reinforcement, such as sugar rewards (Hendel et al., 2005; Gerber and Hendel, 2006). Upon pairing of an odour with sugar (fructose), larvae show enhanced preference towards the odour as a result of learned behaviour (Saumweber et al., 2011). We employed an established standardised behavioural assay to analyse learning and memory in larvae (Widmann et al., 2018). The learning ability as reported by the PI differed significantly between groups using an unpaired Student’s *t*-test [mean PI: w*^1118^*: 0.273 ± 0.028, TPI: 0.055 ± 0.027, *p* < 0.0001 (*n* = 27–37) Figure 8A]. Assessing learning abilities using a one-sample Wilcoxon test compared to the theoretical value 0, revealed that TPI dysfunction renders larvae unable to learn (*p* = 0.0677), whereas WT larvae showed substantial odour associative learning (*p* < 0.0001). In order to test whether changes in innate preference for AM was potentially masking the difference in learning abilities, we determined odour preference in both genotypes to assess the innate attraction. The innate preference was similar in both genotypes [innate preference AM: w*^1118^*: 0.262 ± 0.071, TPI: 0.200 ± 0.055, *p* = 0.493 (*n* = 6 each) Figure 8B], thus not contributing to associative learning.

### Longevity

Measurements of life span provides information about accelerated aging due to various forms of neuronal dysfunction (Linford et al., 2013). TPI mutant flies showed a drastically reduced life span compared to wild type flies with medial survival rates were reduced from 60 days in w*^1118^* to 40 days in wstd^1^ animals [*p* = 0.0047, Log-rank (Mantel–Cox test) Supplementary Figure 1] suggesting that the impaired activity of TPI impacts on neuronal health over the lifetime.

## Discussion

Our studies were set up to investigate functional effects on synaptic transmission caused by impaired TPI activity. TPI function has been studied in *Drosophila* and rodent models, and a human disease associated with a missense mutation of TPI results in severe glycolytic enzymopathy, the only mutation which is lethal, frequently in early childhood (Orosz et al., 2009). Mutant flies expressing impaired TPI (*sugarkill* or *wasted away* [wstd^1^]) exhibit phenotypes showing analogous symptoms and characteristics to those of human TPI deficiency, including progressive locomotor impairment, vacuolar neuropathology and severely reduced life span (Celotto et al., 2006; Gnerer et al., 2006). In addition, a TPI dysfunction has been reported in AD where the protein is 3-nitrotryrosinated rendering it less active (Tajes et al., 2014b). 3-NT is a NO-mediated post-translational modification associated with prolonged and enhanced neuroinflammation and upregulation of inducible NO synthase (iNOS) expression (Heneka and Feinstein, 2001; Gu et al., 2010; Steinert et al., 2010; Bradley and Steinert, 2016; Nakamura and Lipton, 2020). In any case where TPI activity is compromised, either genetically or post-translationally, there is an accumulation of the metabolites of this isomerase, DHAP and methylglyoxal, which induce protein glycation (Ott et al., 2014).

**FIGURE 9:**
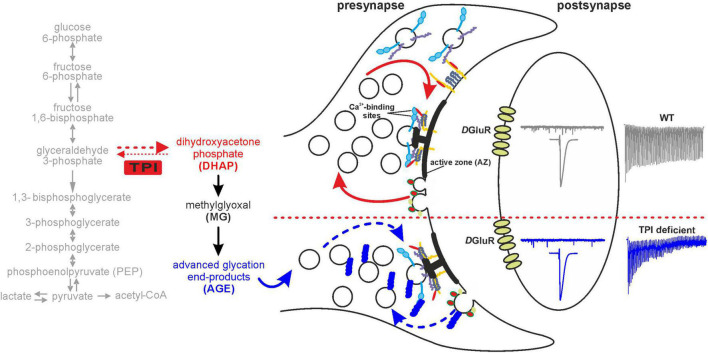
Schematic illustration of TPI-mediated glycation signalling at the synapse. The schematic shows the effects of TPI function mediating the interconversion of GAP to DHAP. DHAP itself is spontaneously converted to the glycating compound methylglyoxal which is responsible for generating advanced glycation end products. Our data is compatible with a model by which presynaptic proteins undergo enhanced glycation as a consequence of TPI deficiency. Presynaptic proteins involved in transmitter release or recycling are potential targets and their dysregulation may impact upon vesicular release. In particular, under high frequency stimulation this may manifest itself in a slowed recovery of vesicle pool depletion and detected as greater depletion during higher frequency trains, whereas single and low frequency activity is unaffected. Interestingly spontaneous frequency of vesicular release is also reduced following TPI deficiency. Potential target proteins include syntaxin [yellow]/Munc-18 [red], SNAP-25 [dark purple], synaptobrevin [purple], synaptotagmin [blue] which facilitate vesicle fusion or for example clathrin [red/green], synaptojanin [yellow] or dynamin located on endocytosing vesicles.

Our data provides novel findings of functional effects of TPI impairment (wstd^1^) at the synaptic level. We found an increased vesicular depletion during trains of high activity (>30 Hz) which may be caused by a reduced replenishment of releasable pools. We further showed that the rate of spontaneous release is reduced in wstd^1^ animals, both findings pointing toward a dysregulation of vesicular recycling, hallmarks of synaptopathologies (Figure 9). The data is consistent with a computational model showing that the TPI mutation causes an accelerated decline in available vesicles during higher frequency stimulation trains with limited replenishment due to reduced vesicle recovery. It is likely, as a result of the overserved neuronal dysfunction, that the TPI mutation induces impairments in learning and memory at larval stages and is responsible for a reduced life span in adulthood possibly due to accelerated aging processes.

Synaptic vesicular release forms the basis of neuron-to-neuron or neuron-to-muscle (NMJ) communication. A multitude of signalling steps is involved in vesicle endo- and exocytosis (for review, Gundelfinger et al., 2003; Sudhof, 2004). *Drosophila* in particular, presents an excellent model system to study synaptic release mechanisms, and we and others have utilised this model to characterise various forms of synaptic plasticity and dysfunction in diseases models (Kuromi and Kidokoro, 2005; Steinert et al., 2006; Weyhersmuller et al., 2011; Robinson et al., 2014, 2017, 2018; Bohme et al., 2019; Spiers et al., 2019). The presynaptic release machinery at the *Drosophila* NMJ includes highly conserved endo- and exocytosis pathways and vesicle recruitment from different internal pools, and our data points towards an effect of TPI dysfunction on these release pathways without affecting the release probability (Figure 3), NMJ morphology (Figure 6) or bioenergetics (Celotto et al., 2006; Gnerer et al., 2006). Under low activity (1–20 Hz stimulation), TPI deficiency does not impact on release, however, at higher stimulus frequencies (>30 Hz), we detected an enhanced depletion over a 1,000 ms period followed by a slowed recovery (Figure 9). Similar phenotypes have been reported under conditions where endophilin dysfunction causes an inability to retrieve synaptic membranes accompanied by a depletion of vesicles from the bouton lumen (Verstreken et al., 2002), and block of clathrin-mediated endocytosis, which is the major pathway by which vesicles are regenerated. Interestingly, this mutant also leads to a reduction in spontaneous release frequencies further supporting the notion of an endocytosis effect. Further evidence for synaptic dysfunction stems from studies reporting that glycated tau binds to synaptic vesicles via its N-terminal domain which interferes with presynaptic functions, including synaptic vesicle mobility and release rate resulting in compromised neurotransmission in fly and rat neurons (Zhou et al., 2017). Endocytosis is regulated by an orchestrated activity of numerous vesicular and other presynaptic proteins, however, our data does not allow a firm conclusion as to which specific step in endocytosis may be affected. Nonetheless, there is evidence that a knock-out of palmitoyl-protein thioesterase 1 causes strong deficit in endocytosis (Aby et al., 2013). Palmitoyl-protein thioesterase 1 is responsible for the removal of a palmitate group from its substrate proteins, which may include presynaptic proteins like Synaptosomal-Associated Protein 25 (SNAP-25), cysteine string protein, dynamin and synaptotagmin and the authors suggest that an inefficient de-palmitoylation compromises dysfunction of proteins involved in endocytosis.

The aforementioned important post-translational modification, glycation, becomes a crucial and overwhelmingly cytotoxic process following reduction of TPI function, and resulting glycation of proteins may impact on their localisation and function (Ott et al., 2014). Protein glycation has been implicated in neuronal dysfunction, for example glycated α-synuclein exhibits impaired binding to synaptic-like vesicles leading to an inability to facilitate fusion (Uceda et al., 2022). Enhanced glycation also facilitates other neurodegenerative signalling cascades and can cause tau pathology and memory loss (Li X. H. et al., 2012; Guerrero et al., 2013; Alquezar et al., 2020). TPI function is compromised in AD (Tajes et al., 2014b) as a result of its 3-nitrotyrosination, the reported increase in glycation may also represent a mechanism to induce or aggravate synaptopathologies. Although glycation and related AGE formation has been implicated in causing suppression of hippocampal and cortical synaptic plasticity (Zhang et al., 2014; Criscuolo et al., 2017), altering glutamatergic transmission (Chegao et al., 2022) inducing memory deficits (Li X. H. et al., 2012), direct protein targets involved in vesicle recycling have not yet been reported.

Together, our study investigated the effects of a TPI deficiency on synaptic activities and the detected alterations in vesicular release and replenishment may implicate an enhanced glycation of synaptic proteins which can lead to protein dysfunction, and result in the observed synaptic phenotypes. Although future studies will have to identify protein targets, our data presents a novel synaptic phenotype connecting TPI deficiency impairment with synaptic dysfunction, a mechanism which may be involved in aggravating neurodegeneration.

## Data availability statement

The raw data supporting the conclusions of this article will be made available by the authors, without undue reservation.

## Author contributions

AS performed and analysed electrophysiological, ICC, and longevity studies and helped writing the manuscript. OC, AR, ES, and SA performed and analysed locomotor and odour learning studies. BG performed the computer modeling. JS conceptualised the project, analysed the data, and wrote the manuscript. All authors contributed to the article and approved the submitted version.

## Supplementary material

Supplementary Figure 1 wstd^1^ Drosophila exhibit a reduced life span. Longevity was recorded at 25°C with a 12 h light-dark cycle. Median life spans were 40 days and 60 days for wstd^1^ (*n* = 90) and w*^1118^* (*n* = 90), respectively [Log-rank (Mantel-Cox) test, **p* < 0.05].
