# Supplementary figures and images for: Triose-phosphate isomerase deficiency is associated with a dysregulation of synaptic vesicle recycling in Drosophila melanogaster
Source: Front Synaptic Neurosci. 2023 Feb 28;15:1124061. doi: 10.3389/fnsyn.2023.1124061 (PMC10011161; doi:10.3389/fnsyn.2023.1124061)

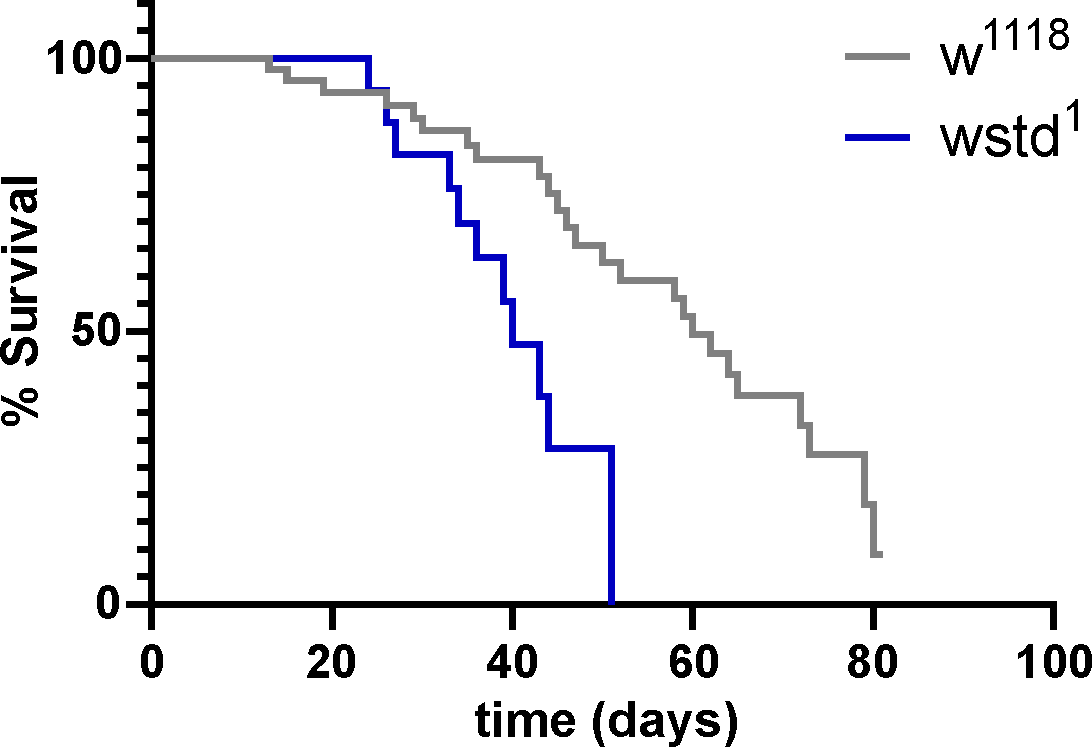

Supplement: Supplementary Figure 1 — wstd1 Drosophila exhibit a reduced life span. Longevity was recorded at 25°C with a 12 h light-dark cycle. Median life spans were 40 days and 60 days for wstd1 (n = 90) and w1118 (n = 90), respectively [Log-rank (Mantel-Cox) test, *p < 0.05]. [file Image_1.TIF]
